# Interspecific variation in gut microbiome diversity across the Etosha National Park herbivore community

**DOI:** 10.1371/journal.pone.0333639

**Published:** 2025-10-09

**Authors:** Rylee Jensen, Erin A. McKenney, James C. Beasley, Claudine C. Cloete, Madeline Melton, Diana J. R. Lafferty

**Affiliations:** 1 Department of Biology, Northern Michigan University, Marquette, Michigan, United States of America; 2 Department of Applied Ecology, North Carolina State University, Raleigh, North Carolina, United States of America; 3 Warnell School of Forestry & Natural Resources, University of Georgia Athens, Athens, Georgia, United States of America; 4 Ministry of Environment, Forestry and Tourism, Etosha Ecological Institute, Okaukuejo, Namibia; University of the Faroe Islands: Frodskaparsetur Foroya, FAROE ISLANDS

## Abstract

The community of microbes in the gastrointestinal tract of mammals, known as the gut microbiome (GMB), plays a critical role in host ecology and evolution. GMB variation is modulated by both host physiology and environmental conditions experienced by the host. Here we characterized the GMBs of 11 free-ranging large herbivore species inhabiting Etosha National Park, Namibia. We examined how intrinsic (i.e., sex, gut morphology, feeding guild) and extrinsic (i.e., geographic zone, waterhole site) factors influenced GMB diversity and community structure within and across herbivore species. We extracted DNA from herbivore fecal samples (n = 312) and amplified the 16s rRNA gene region to identify bacterial taxa. We defined core bacterial taxa as those present at ≥1% relative abundance in ≥50% of the samples from each species. Within bovid species, the core phylum *Verrucomicrobiota* and the core genera *RF39*, *Alistipes*, *Christensenellaceae_R-7 group*, and *NK4A214* were significantly different in abundance across geographic zones. Microbial richness was significantly greater in female than male eland, and we detected sex-specific differences in *Christensenellaceae_R-7 group* across all herbivores and *P-251-O5* within gemsbok. Mean Bulla evenness was higher in ruminants than nonruminants and differed significantly between giraffes and impala. Elephants also showed a significant correlation between unweighted UniFrac distance and geographic distance between sample locations. By identifying baseline core microbial abundance and occurrence data for this herbivore community, wildlife managers can incorporate long-term GMB monitoring to track microbial shifts in host species over time.

## Introduction

Microbial communities inhabiting the gastrointestinal tract of mammals, collectively known as the gut microbiome (GMB), play a critical role in all facets of host ecology and evolution [1–3]. For instance, the GMB performs a multitude of physiological services for the host such as conferring enzymatic processes essential for nutrient uptake and digestion [1], triggering immune responses [4], initiating hormone releases to regulate metabolism [5], activating stress responses [6], resisting pathogenic invasion [7], and even influencing mood and anxiety [8,9]. GMB diversity (i.e., richness and composition) and community structure (i.e., relative abundance), jointly referred to as a GMB profile, are influenced by multiple factors intrinsic to the host including phylogeny [10–12], life stage [13], sex [14], stress [6,15], diet [16–18], and physiology [19].

GMB profiles are also fundamentally linked to the host’s environment [20,21], and thus sensitive to habitat perturbations [17,22,23] and stressors like long-term drought [24] and arid environments [25]. In fact, variation in extrinsic conditions can influence GMB composition [20] and community structure [6] by changing the type and abundance of available foods. In more extreme cases, changes to an otherwise stable ecosystem may induce dysbiosis within the host through long-term microbial community shifts and loss of important physiological functions [23,26]. For example, reduced dietary diversity stemming from anthropogenic habitat degradation resulted in a perturbation in the GMB and an overall weaker immune system in threatened and endangered primate species in Central America [23] and Africa [22,26]. There are also numerous examples of environmental contamination and climate change impacts permanently altering GMB composition within a variety of host species [27].

Past studies examining the effects of environmental conditions on host GMBs have often used only one or two host species as biological models. However, studies that incorporate multiple host species from the same community [10,12,28] can potentially examine how the GMB profiles of hosts with different physiologies, dietary niches, and evolutionary histories are impacted by the same set of intrinsic and extrinsic factors. As a result, interspecific differences in GMB profiles may be driven by host species that are naturally more sensitive to microbial fluctuations than others [28] or prevalent microbial taxa that serve specialized functions for their associated hosts [19,29]. Thus, there is a need to examine trends in abundance and composition of specific microbial taxa within and across a range of host species.

The diverse community of free-ranging herbivores inhabiting Etosha National Park (ENP) in northern Namibia provides an excellent model system in which to test the strength of intrinsic and extrinsic drivers of GMB profiles among species. First, the ENP herbivore community includes species with differing gut morphologies (i.e., ruminants and nonruminants), feeding guilds (i.e., browsers, grazers, and mixed foragers), diets, body sizes, and phylogenetic histories, enabling us to analyze how multiple intrinsic traits shape GMB profiles across species. Unique patterns of GMB variation can be elucidated at different host taxonomic scales [30–32], as demonstrated with African bovid species [32], many of which inhabit ENP. Additionally, the extreme semiarid environment of ENP lies across a prominent west-to-east precipitation gradient with distinct vegetative ecoregions [33,34], allowing us the unique opportunity to examine how GMB profiles may reflect extrinsic variation associated with the precipitation-dependent nutritional landscape of ENP. Furthermore, due to limited water availability during the dry season, herbivore species often congregate in large densities at natural and artificial waterhole sites scattered across ENP. Resource-sharing and proximity to conspecifics at waterholes could alter individual GMB communities through indirect microbial transmission, as demonstrated in other free-ranging mammal species [35–37].

Our objective was to evaluate how host GMBs are modulated by coalescing intrinsic and extrinsic factors among a diverse community of mammalian herbivores in ENP. Identifying trends in GMB variation (e.g., microbial membership and diversity) across herbivore species can provide insight into which microbial taxa are conserved across hosts and thus may be collectively important to ENP’s herbivore community. Additionally, analyzing GMB variation within herbivore species could infer specialized microbial functions that contribute to host fitness and survival. Semiarid environments such as ENP can shape GMB profiles in a variety of ways. For example, limited resource availability in drier ecosystems can lead to hosts consuming more varied diets with altered nutritional compositions, which can alter microbial diversity [12]. Osborne et al. [25] postulates that arid-adapted host species may be particularly important for providing refugia in arid environments. Previous studies have characterized GMB profiles of conspecific hosts in other regions of Africa such as Maasai Mara National Reserve [32] and Laikipia [12] in Kenya. While we did not directly compare our results to these studies, we offer an alternative perspective about the GMB profiles of some of the same herbivore species inhabiting the harsher, more resource-strained environment of ENP [38].

Among other extrinsic factors that could impact herbivore GMB profiles, we hypothesized that resource-sharing among different herbivore species at waterhole sites would manifest as intraspecific differences in GMB profiles across different zones and waterhole sites in ENP. We also hypothesized that herbivore GMB profiles would exhibit significant differences in GMB diversity and community structure associated with sex, gut morphology, feeding guild, and herbivore species ID.

## Materials and methods

### Study area

**Fig 1:**
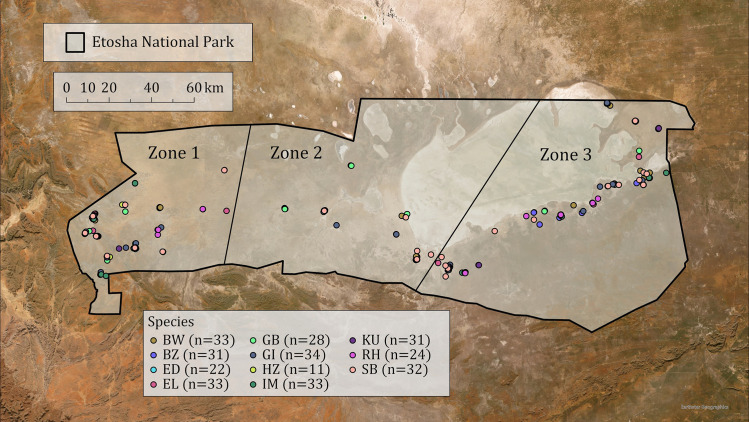
Map of herbivore GPS points in Etosha National Park in northern Namibia. GPS points of collected fecal samples from 11 herbivore species are represented by colored points throughout the park, with each color corresponding to a specific species. “BW” represents blue wildebeest (*Connochaetes taurinus*), “BZ” represents Burchell’s/plains zebras (*Equus quagga burchellii*), “ED” represents eland (*Taurotragus oryx*), “EL” represents African elephants (*Loxodonta africana*), “GB” represents gemsbok/oryx (*Oryx gazella*), “GI” represents Angolan giraffes (*Giraffa camelopardalis angolensis*), “HZ” represents Hartmann’s/mountain zebras (*Equus zebra hartmannae*), “IM” represents black-faced impala (*Aepyceros melampus petersi*), “KU” represents kudu (*Tragelaphus strepsiceros*), “RH” represents red hartebeest (*Alcelaphus buselaphus*), and “SB” represents springbok (*Antidorcas marsupialis*). Map image is the intellectual property of Esri and is used herein under license. Copyright © 2023 Esri and its licensors. All rights reserved.

Etosha National Park (ENP) is a semiarid environment in northern Namibia that encompasses roughly 22,270 square kilometers (Fig 1). ENP is named after the Etosha salt pan, which is the largest salt pan on the continent. The park receives highly variable periods of precipitation [34,38], with droughts often lasting more than a decade; the park has been experiencing a drought period since 2013 [38]. The western region of ENP receives considerably less precipitation than the east [34], which in turn affects vegetative communities across the park. West and north of the Etosha pan is a dry, sparse shrubland ecoregion composed of species such as moringa (*Moringa oleifera*) and mopane (*Colophospermum mopane*). The northeast section of the park is Kalahari woodlands, which feature clay-like soils and tree species like camelthorn (*Vachellia erioloba*) and Angola teak (*Pterocarpus angolensis*). South and east of the Etosha pan is dominated by Karstveld woodlands with trees such as tamboti (*Spirostachys africana*) and acacia (*Acacia spp*.), and various shrubs (e.g., *Suaeda articulate*). Surrounding the Etosha pan are perennial, halophilic grasses such as salt grass (*Sporobolus spicatus*) and steekgrass (*Odyssea paucinervis*) [33,34]. The ENP perimeter is fenced, though this does not completely restrict herbivore movement outside of the park. The landscape includes many waterhole sites that attract large wildlife congregations including a variety of mammal and bird species.

### Fecal collection

ENP was divided into three sampling zones to ensure that representative samples of each species were obtained across the ENP precipitation gradient, which increases from west to east. Zone 1 encompasses the westernmost region and Zone 3 encompasses the easternmost region. The line between Zones 2 and 3 was drawn toward the northeast to include a vegetation strip west of the line on the northern boundary of ENP. Fecal samples (n = 312) were noninvasively collected from 11 herbivore species across ENP from July-September 2022, encompassing much of the dry season. Sampled species included African elephants (*Loxodonta africana*), Angolan giraffes (*Giraffa camelopardalis angolensis*), Burchell’s/plains zebra (*Equus quagga burchellii*), Hartmann’s/mountain zebra (*Equus zebra hartmannae*), kudu (*Tragelaphus strepsiceros*), common eland (*Taurotragus oryx*), springbok (*Antidorcas marsupialis*), black-faced impala (*Aepyceros melampus petersi*), gemsbok/oryx (*Oryx gazella*), red hartebeest (*Alcelaphus buselaphus*), and blue wildebeest (*Connochaetes taurinus*; Table 1). Sample collection was balanced evenly across the three zones and additional effort was made to ensure comparable sampling of males and females for each species. Animals were observed from vehicles along roads and near waterholes, and fecal samples were collected within 15 minutes after defecation for most species, and within 30 minutes for elephants. Samples were only collected from individuals for whom sex and approximate age class could be determined. Samples were then preserved in PERFORMAbiome (PB-200) animal gut microbial DNA collection tubes (DNA Genotek Inc. Kanata, Canada) and labeled with essential metadata and covariate information including species, age, sex, GPS location, and date/time.

**Table 1:** Classification of African herbivore community in Etosha National Park, Namibia.

Species	Scientific Name	Family	n	Sex	Feeding Guild	Gut Morphology
African elephant	*Loxodonta africana*	Elephantidae	33	F = 19, M = 14	Mixed forager	Nonruminant
Angolan giraffe	*Giraffa camelopardalis angolensis*	Giraffidae	34	F = 18, M = 16	Browser	Ruminant
Burchell’s zebra	*Equus quagga burchellii*	Equidae	31	F = 15, M = 16	Grazer	Nonruminant
Hartmann’s zebra	*Equus zebra hartmannae*	Equidae	11	F = 5, M = 6	Grazer	Nonruminant
Common eland	*Taurotragus oryx*	Bovidae	22	F = 10, M = 12	Mixed forager	Ruminant
Gemsbok/oryx	*Oryx gazella*	Bovidae	28	F = 15, M = 13	Grazer	Ruminant
Kudu	*Tragelaphus strepsiceros*	Bovidae	31	F = 16, M = 15	Browser	Ruminant
Red hartebeest	*Alcelaphus buselaphus*	Bovidae	24	F = 12, M = 12	Grazer	Ruminant
Blue wildebeest	*Connochaetes taurinus*	Bovidae	33	F = 17, M = 16	Grazer	Ruminant
Black-faced impala	*Aepyceros melampus petersi*	Bovidae	33	F = 18, M = 15	Mixed forager	Ruminant
Springbok	*Antidorcas marsupialis*	Bovidae	32	F = 16, M = 16	Mixed forager	Ruminant

Common and scientific names, taxonomic family, sample size, sex, feeding guild, and gut morphology of 11 African herbivore species sampled in Etosha National Park, Namibia. Species are listed according to evolutionary relationships, from most distantly related at the top to closely related at the bottom. Numbers in the “Sex” column refer to number of fecal samples collected from females (F) and males (M) of each species.

### Laboratory methods

All fecal samples were imported from Windhoek, Namibia into the United States via the Chicago Port Authority in accordance with both Namibian Ministry of Environment, Forestry and Tourism (MEFT; AN202208007) and United States Department of Agriculture (USDA) Animal and Plant Health Inspection Service (APHIS) research permits (639-22-245-02893) and U.S. Convention on International Trade in Endangered Species of Wild Fauna and Flora (CITES) import permit (23US41867E/9). An International Animal Care and Use Committee (IACUC) written exemption was obtained from Northern Michigan University (NMU) for the use of fecal samples.

At NMU’s Wildlife Ecology and Conservation Science (WECOS) Lab, we extracted DNA using DNEasy PowerSoil Pro kits (QIAGEN) adapted from QIAGEN’s established protocols. Feces naturally disintegrated in the PB-200 tube buffers as they were stored at ambient temperatures; rather than using 250 mg of solid stool as the protocol calls for, we vortexed each tube for several seconds and then pipetted 200 µL of liquid fecal material to ensure we extracted DNA from representative samples. We spectrophotometrically quantified nucleic acid concentrations (using a factor of 50 ng/µL) for each sample using a Nanodrop-2000c (ThermoFisher Scientific, Waltham, Massachusetts, USA). We stored DNA extracts at −62.22 °C (−80°F) after processing was complete. We then aliquoted samples in equimolar ratios (20 µL) and shipped all 312 aliquots to Argonne National Laboratory (ANL) in Chicago, IL. PCR amplification of the V4 gene region of the bacterial 16S rRNA gene was amplified (as described by Caporaso et al. [39]) using the forward primer 515F (sequence GTGYCAGCMGCCGCGGTAA) and reverse primer 806R (sequence GGACTACNVGGGTWTCTAAT) and subjected to 2 × 150 paired-end reads on Illumina’s MiSeq platform. ANL uses a standard laboratory protocol that includes negative PCR controls in every plate; pooling and sequencing only proceeds if no DNA is amplified from negative PCR controls.

### Bioinformatic analyses

We imported raw sequences into the bioinformatics visualization software Quantitative Insights into Microbial Ecology (QIIME2; version 2023.7) [40] via the miniconda3 platform. After joining sequences, quality-filtering, and demultiplexing using default values, we denoised sequences using the Divisive Amplicon Denoising Algorithm (DADA2) QIIME2 plugin (version 1.26.0 via *bioconductor*) [41] and truncated sequence lengths to 150 bp. We then used a pre-trained Naïve Bayes classifier [42,43] to assign microbial taxonomic classification to the genus level with the SILVA 99 reference database (version 138.1) [44,45] and filtered sequences to remove Eukaryota, mitochondria, chloroplasts, and unassigned bacterial taxa. We used scaling with ranked subsampling (SRS) [46] normalization in RStudio (version 4.3.2) [47] with a C_min_ value of 5,244 to preserve all 312 samples, which retained a total of 27,340 sequences.

### Statistical analyses

We completed all subsequent statistical analyses and visualizations in RStudio (version 4.3.2) [47]. We used the package *qiime2R* (version 0.99.6) [48] to import sequence and taxonomic data and then converted them to *phyloseq* objects (version 1.46.0) [49]. To determine potential differences in microbial community compositions among herbivore species, we analyzed GMB profiles at both the phylum and genus levels to assess the impacts of intrinsic and extrinsic drivers that may manifest at different microbial taxonomic scales, as previously demonstrated in bamboo specialists [16].

#### Community structure.

We first identified “major” microbial taxa present in ≥1% relative abundance across all herbivore samples. Next, we identified “core” microbial taxa, defined as major microbial taxa that occur within a certain threshold of herbivore samples that we designated *a priori*. Specifically, we identified core microbial taxa that occurred in ≥90%, ≥ 80%, ≥ 75%, and ≥50% of all 312 herbivore samples. Using a combination of abundance and occurrence analysis methods more accurately identifies core microbial membership than using either method alone [50] and provides a robust representation of selection for specific microbial taxa within host species and clades [51]. We then identified herbivore species-specific core microbial taxa using the same criteria (e.g., major microbial taxa that occurred in ≥90%, ≥ 80%, ≥ 75%, and ≥50% of samples from each host species). Comparing the core microbial taxa presence along the spectrum from the most stringent (90%) to the least stringent (50%) thresholds may elucidate potential functional roles and important selective constraints unique to different herbivore host species. After examining herbivore species-specific differences in GMB profiles, we elected to use the ≥ 50% core microbial level for all subsequent analyses and comparisons across herbivore samples.

We analyzed core microbial taxa detected in ≥50% of individuals across and within herbivore species as opposed to the ≥ 90%, ≥ 80%, or ≥75% levels based on assumptions of microbial functionality in host species and limitations with taxon sample sizes. First, if particular microbial taxa are still present in ≥50% samples from some herbivore species but not others, it may be reasonable to infer that factors beyond phylogeny influence diversity and community structure in the GMBs of those herbivores (e.g., host environment, disease, etc.). Second, while setting a core threshold below 50% may reveal additional host-associated microbial taxa within herbivore species, it is less likely that less prevalent microbial taxa have evolved functions that specifically complement host physiology, and there is a greater likelihood of mistaking natural individual variation for taxa adapted to the host species. Lastly, sample size was a limiting factor for microbial taxon-specific analyses, especially for identifying core microbial taxa present in some host species (e.g., Hartmann’s zebra with n = 11 samples).

We compared fecal microbial communities based on host species and three additional intrinsic traits: sex, gut morphology (ruminants and nonruminants), and feeding guild (browsers, grazers, and mixed foragers), confirmed by existing literature [32,52,53] (Table 1). We ultimately classified gemsboks’ feeding guild as grazers [53,54], but also ran analyses with gemsbok classified as mixed foragers [52,55] and browsers [56] to reflect disagreement in the literature. We also included a set of two categorical covariates to determine the effects of extrinsic conditions on GMB profiles based on where samples were collected: geographic zone and waterhole site (based on the named waterhole to where the sample was collected). We used a cutoff distance of 5 km to assign herbivore samples to specific waterhole sites.

We first tested assumptions of normality within each of the six intrinsic and extrinsic variables (i.e., herbivore species ID, sex, gut morphology, feeding guild, zone, and waterhole site) with Shapiro-Wilk tests using the *RVAideMemoire* package (version 0.9-83-7) [57] and by checking residual plots. We tested for homogeneity of variance with Levene’s test using the *car* package (version 3.1–2) [58]. We then tested for differences in the mean abundance of each core microbial taxon present in ≥50% of all herbivore samples using separate analyses for each of the six categorical variables. For variables that included three or more groups (e.g., feeding guild), this was done with one-way analysis of variance (ANOVA) tests with the package *stats* (version 4.3.2) [59], bootstrapped one-way ANOVA tests with the package *WRS2* (version 1.1–5) [60], and permutation one-way ANOVA tests with the package *wPerm* (version 1.0.1) [61]. For variables that included two groups (e.g., gut morphology), we used unpaired two-tailed t-tests (including bootstrapped and permutation t-tests for groups that were not normally distributed and with lower sample sizes) and Kruskal-Wallis rank sum tests using the packages *stats* and *MKinfer* (version 1.1), respectively [62]. We only analyzed waterhole sites where ≥10 samples were collected, which included 13 total sites comprising 210 herbivore samples. We only used these 210 samples to determine the specific effect of waterhole as an extrinsic variable; we included all 312 samples in analyses for all other intrinsic and extrinsic variables. For each of these analyses, we followed significant one-way ANOVA tests with a Tukey’s post-hoc test for pairwise comparison of means among groups, while significant bootstrapped one-way ANOVAs were followed by a bootstrapped version of a post-hoc test. We performed these analyses across all 11 herbivore species first, then repeated each test with a subset of the data comprising the seven bovid species (Table 1). Additionally, we analyzed any microbial taxa that were present in ≥50% of individuals within some (but not all) herbivore species, across only those respective herbivore species. We then adjusted all statistically significant p-values with False Discovery Rate (FDR) correction [63] with the *stats* package to account for the expected proportion of false positive tests.

#### Alpha diversity.

We quantified nine alpha diversity indices for each herbivore sample (n = 312) to determine if any significant differences in diversity arose within each of the six categorical variables when we calculated richness and evenness separately and then together in a single metric. We used the *picante* package (version 1.8.2) [64] to calculate species richness (SR) and Faith’s Phylogenetic Diversity (hereafter, Faith’s PD), which measures richness while accounting for phylogenetic relationships among microbial taxa [65]. We used the *microbiome* package (version 1.24.0) [66] to calculate five evenness indices: Camargo [67], Pielou [68], Simpson [69], E_var_ [70], and Bulla [71]. We also used the *microbiome* package to calculate Shannon diversity [72] and inverse Simpson’s diversity [69]. Both Shannon and inverse Simpson’s indices measure richness and evenness in a single metric, though Shannon diversity places greater weight on rarer species.

To test for significant differences in mean alpha diversity, we ran separate analyses of each alpha diversity index for each of the six categorical variables. For variables that included three or more groups, we ran one-way ANOVA tests (including bootstrapped one-way ANOVAs for groups that were not normally distributed) with the packages *stats* and *WRS2*. For variables that included two groups, we used two-tailed t-tests (including bootstrapped t-tests for groups that were not normally distributed) and Kruskal-Wallis rank sum tests (for groups with low sample sizes) using the packages *stats* and *MKinfer*. We performed these analyses for all 11 herbivore species first, then repeated each test for the seven bovid species (Table 1). P-values were once again adjusted with FDR correction.

#### Beta diversity.

We calculated two beta diversity indices to measure pairwise distance among all herbivore samples (n = 312): weighted and unweighted UniFrac distances. While both UniFrac distances account for phylogenetic branch length, weighted UniFrac incorporates the relative abundance of each taxon, whereas unweighted UniFrac focuses solely on the presence/absence of microbial membership [73]. UniFrac distances both account for phylogenetic branch length unique to each microbial community in a given pairwise comparison [73]. We created distance matrices of each index to compare against the six intrinsic and extrinsic variables with a permutational multivariate analysis of variance (PERMANOVA) using the *vegan* package (version 2.6–4) [74]. We also tested whether herbivore species exhibited significantly different intraspecific variation across the ENP landscape. To do this, we first calculated geographic distance between all pairwise herbivore samples (n = 97,344) using the package *geosphere* (version 1.5–18) [75]; we used Haversine distance, which assumes a spherical earth and ignores ellipsoidal effects. We then subset the overall geographic distance matrix into individual distance matrices for all 11 herbivore species and created herbivore species-specific weighted and unweighted UniFrac distance matrices. We compared herbivore species-specific beta diversity matrices against the herbivore species-specific geographic distance matrices with Mantel tests using the non-parametric Spearman rank sum method (2000 permutations) [76].

## Results

Between July and September 2022, 312 fecal samples were collected with comparable division between males and females of each species as well as across the three Zones established across ENP for most species (Table 1). Hartmann’s zebra samples were only collected in Zone 1 and no eland samples were collected in Zone 2. Although age class of sampled individuals was initially documented in the field, we did not consider this variable for statistical analysis due to small sample sizes for subadult (n = 11) and immature (n = 3) animals and therefore the total number of samples reflects adults from each species.

### Community structure

**Fig 2:**
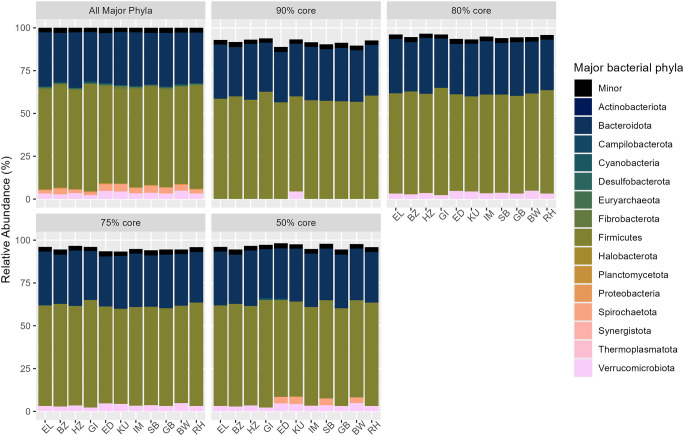
Core bacterial phyla in Etosha National Park’s herbivore community. Comparison of 15 major bacterial phyla (present at >1% abundance) to core phyla, defined as phyla present in ≥90%, ≥ 80%, ≥ 75%, and ≥50% of all samples within each herbivore species (n = 11) sampled in Etosha National Park, Namibia. Minor category includes all phyla present at <1%. Herbivore species identification is represented as “EL” for African elephants (*Loxodonta africana*), “BZ” for Burchell’s/plains zebra (*Equus quagga burchellii*), “HZ” for Hartmann’s/mountain zebra (*Equus zebra hartmannae*), “GI” for Angolan giraffes (*Giraffa camelopardalis angolensis*), “ED” for common eland (*Taurotragus oryx*), “KU” for kudu (*Tragelaphus strepsiceros*), “IM” for black-faced impala (*Aepyceros melampus petersi*), “SB” for springbok (*Antidorcas marsupialis*), “GB” for gemsbok/oryx (*Oryx gazella*), “BW” for blue wildebeest (*Connochaetes taurinus*), and “RH” for red hartebeest (*Alcelaphus buselaphus*). Herbivore species are listed according to phylogenetic relatedness, with the most evolutionarily separated species on the left and closely-related species on the right. There were two phyla (*Bacteroidota* and *Firmicutes*) present at the ≥ 90% core level and three phyla (*Bacteroidota*, *Firmicutes*, and *Verrucomicrobiota)* present at the ≥ 80%, ≥ 75%, and ≥50% core levels that were shared among all herbivore species. The teal-colored phylum present in the major phyla group and 50% core level in eland, giraffes, and impala is *Cyanobacteria*. The salmon-colored phylum present in the major phyla group and 50% core level in blue wildebeest, eland, kudu, and springbok is *Spirochaetota*.

Of the 27 unique microbial phyla identified across all 312 samples from individual herbivores, there were 15 major phyla (i.e., occurring at ≥1% relative abundance) and five core phyla (i.e., major phyla that are abundant in a specific *a prior* designated percentage of herbivore species samples). The core phyla *Bacteroidota* and *Firmicutes* were present in ≥90%, ≥ 80%, ≥ 75%, and ≥50% of all samples, while *Verrucomicrobiota* were present in ≥80%, ≥ 75%, and ≥50% of all herbivores (Fig 2). Additionally, *Spirochaetota* were detected in ≥50% of individual blue wildebeest, eland, kudu, and springbok, while *Cyanobacteria* were detected in ≥50% of individual eland, giraffe, and impala (Fig 2). There were not enough herbivore species containing *Cyanobacteria* or *Spirochaetota* in ≥50% individuals to compare across waterhole sites. The presence and absence of these five core phyla (at the ≥ 50% core level) within each herbivore species are outlined in S1 Table.

**Fig 3:**
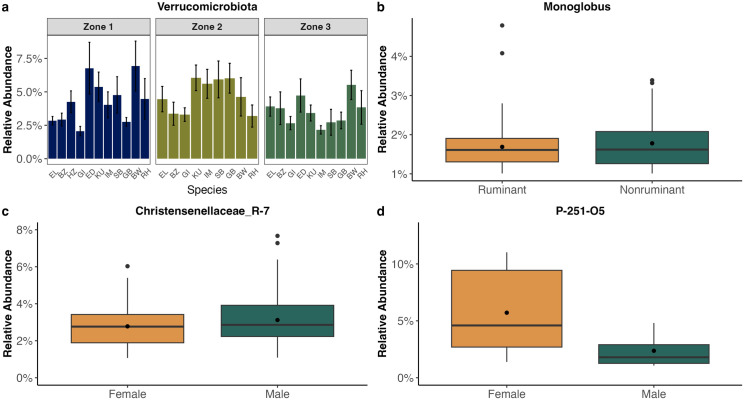
Significant differences in relative abundance of core bacteria based on environmental and physiological factors. Relative abundance of core bacterial phylum (a) and genera (b-d) found in ≥50% of samples per herbivore species that exhibited significant differences associated with specific environmental or intrinsic factors. (a) Among three putatively divided zones of Etosha National Park (ENP) in northern Namibia, the phylum *Verrucomicrobiota* was significantly different between Zones 1 and 2 (bootstrapped one-way ANOVA P = 0.007) and Zones 1 and 3 (bootstrapped one-way ANOVA P = 0.04) across all 11 herbivore species. Herbivore species identification is represented as “EL” for African elephants (*Loxodonta africana*), “BZ” for Burchell’s/plains zebras (*Equus quagga burchellii*), “HZ” for Hartmann’s/mountain zebras (*Equus zebra hartmannae*), “GI” for Angolan giraffes (*Giraffa camelopardalis angolensis*), “ED” for eland (*Taurotragus oryx*), “KU” for kudu (*Tragelaphus strepsiceros*), “IM” for black-faced impala (*Aepyceros melampus petersi*), “SB” for springbok (*Antidorcas marsupialis*), “GB” for gemsbok/oryx (*Oryx gazella*), “BW” for blue wildebeest (*Connochaetes taurinus*), and “RH” for red hartebeest (*Alcelaphus buselaphus*). Herbivore species are listed according to phylogenetic relatedness, with the most evolutionarily separated species on the left and closely-related species on the right. (b) *Monoglobus* was significantly more abundant in nonruminants than ruminants (bootstrapped t-test, corrected P = 0.05). (c) *Christensenellaceae*_*R-7 group* was significantly more abundant in male versus female herbivores (bootstrap t-test, corrected P = 0.04). (d) *P-251-O5* was significantly more abundant in female gemsbok than male gemsbok (permutation t-test, P = 0.05).

Subsequent analyses revealed that there were no significant differences in mean abundance of *Bacteroidota*, *Firmicutes*, *Spirochaetota*, or *Cyanobacteria* among all herbivore species, between sexes, among zones, between ruminants and nonruminants, among feeding guilds, or among waterhole sites. However, mean *Verrucomicrobiota* abundance across all herbivores was significantly different among zones (bootstrap one-way ANOVA F = 3.83, corrected P = 0.04, 2000 bootstraps), specifically between Zones 1 and 3 (P = 0.04) and Zones 1 and 2 (P = 0.007; Fig 3a). This result was consistent when mean *Verrucomicrobiota* abundance was analyzed within only bovid species (bootstrap one-way ANOVA F = 3.41, corrected P = 0.04, 2000 bootstraps), exhibiting a significant difference between Zones 1 and 2 only (P = 0.01). There was no difference in mean *Verrucomicrobiota* abundance among all herbivore species, between sexes, between ruminants and nonruminants, among feeding guilds, or among waterhole sites.

**Fig 4:**
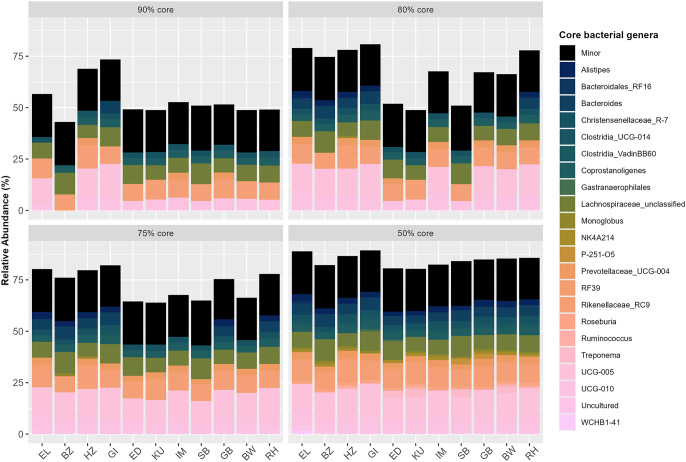
Core bacterial genera in Etosha National Park’s herbivore community. Comparison of four core genera levels, defined as genera that occurs ≥1% relative abundance and is present in ≥90%, ≥ 80%, ≥ 75%, and ≥50% of all samples within each herbivore species (n = 11) sampled in Etosha National Park, Namibia. Minor category includes all genera present at <1%. Herbivore species identification is ordered according to phylogenetic relatedness (distantly-related species on the far left and more closely-related species on the right) and is represented as “EL” for African elephants (*Loxodonta africana*), “BZ” for Burchell’s/plains zebras (*Equus quagga burchellii*), “HZ” for Hartmann’s/mountain zebras (*Equus zebra hartmannae*), “GI” for Angolan giraffes (*Giraffa camelopardalis angolensis*), “ED” for eland (*Taurotragus oryx*), “KU” for kudu (*Tragelaphus strepsiceros*), “IM” for black-faced impala (*Aepyceros melampus petersi*), “SB” for springbok (*Antidorcas marsupialis*), “GB” for gemsbok/oryx (*Oryx gazella*), “BW” for blue wildebeest (*Connochaetes taurinus*), and “RH” for red hartebeest (*Alcelaphus buselaphus*). There were five genera (*Christensenellaceae_R-7 group, Coprostanoligenes, Lachnospiraceae_unclassified* genera*, Rikenellaceae_RC9,* and *UCG-010*) present at the ≥ 90% level, seven genera (the previous five plus *Prevotellaceae_UCG-004* and *UCG-005*) present at the ≥ 80% level, nine genera (the previous seven plus *Bacteroides* and Uncultured genera) present at the ≥ 75% level, and 16 genera (the previous nine plus *Alistipes, Clostridia_UCG-014, Clostridia_VadinBB60, Monoglobus, NK4A214, RF39,* and *Roseburia)* present at the ≥ 50% level that were shared among all herbivore species.

Of the 429 unique microbial genera identified across all 312 individual herbivore samples, we identified 96 major genera (i.e., occurring at ≥1% relative abundance) and 22 core genera (i.e., major genera that are abundant in a certain percentage of herbivore species samples). Five core genera (*Christensenellaceae_R-7 group, Coprostanoligenes, Lachnospiraceae_unclassified* genera, *Rikenellaceae*_*RC9,* and *UCG-010*) were present in ≥90% of all individual herbivores, seven core genera (the previous five plus *Prevotellaceae_UCG-004* and *UCG-005*) were present in ≥80% of all individual herbivores, nine core genera (the previous seven plus *Bacteroides* and Uncultured genera) were present in ≥75% of all herbivore samples, and 16 core genera (the previous nine plus *Alistipes, Clostridia_UCG-014, Clostridia_VadinBB60, Monoglobus, NK4A214, RF39,* and *Roseburia*) were present in ≥50% of all herbivore samples (Fig 4). We identified six additional core genera that were present in ≥50% of individuals within some herbivore species but not others: *Ruminococcus*, *Gastranaerophilales*, *Treponema*, *P-251-O*5, *WCHB1–41*, and *Bacteroidales_RF16* (S1 Table). Interestingly, the latter two genera were only found in ≥50% of elephant samples but not in the majority of individuals from any other herbivore species. The presence and absence of these 22 core genera (at the ≥ 50% core level) within each herbivore species are outlined in S1 Table. There were not sufficient sample sizes across herbivore species containing these latter six species-specific core genera to test for statistically significant differences across waterhole sites.

We discovered significant trends when mean abundance of the 22 core genera present in ≥50% of individual herbivores were analyzed across various intrinsic and extrinsic variables. *Monoglobus* was significantly more abundant in nonruminants than ruminants (bootstrap t-test F = 1.88, corrected P = 0.05, 3000 bootstraps; Fig 3b). *Christensenellaceae*_*R-7 group* was significantly more abundant in males compared to females, both across all herbivores (bootstrap t-test, 2000 reps, corrected P = 0.04; Fig 3c) and within bovid species (bootstrap t-test, 2000 reps, corrected P = 0.04). *P-251-O5* was significantly more abundant in female gemsbok than male gemsbok (permutation t-test, 2000 reps, corrected P = 0.05; Fig 3d).

**Fig 5:**
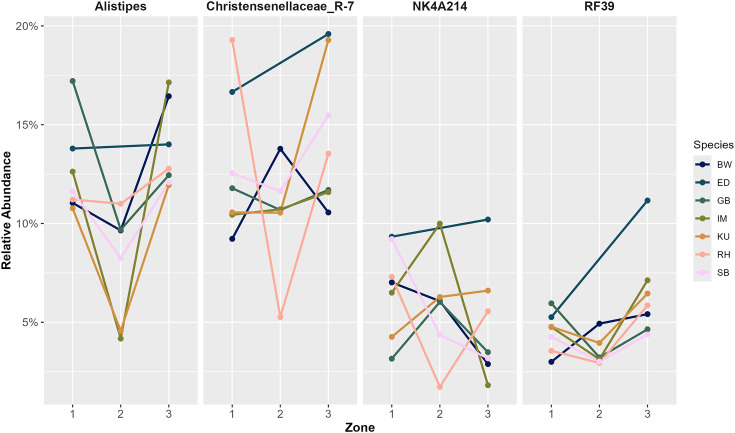
Relative abundance of core bacterial genera fluctuates by geographic zone in Etosha National Park. Relative abundance of four bacterial genera (*Alistipes*, *Christensenellaceae_R-7 group*, *NK4A214*, and *RF39*) among seven African bovid species within three putatively divided zones of Etosha National Park, Namibia. “BW” represents blue wildebeest (*Connochaetes taurinus*), “ED” represents common eland (*Taurotragus oryx*), “GB” represents gemsbok/oryx (*Oryx gazella*), “IM” represents black-faced impala (*Aepyceros melampus petersi*), “KU” represents kudu (*Tragelaphus strepsiceros*), “RH” represents red hartebeest (*Alcelaphus buselaphus*), and “SB” represents springbok (*Antidorcas marsupialis*). No eland samples were collected in Zone 2. *Alistipes* abundance was significantly different between Zones 1 and 2 (bootstrapped ANOVA, corrected P = 0.007), genera identified to the family *Christensenellaceae*_*R-7 group* were significantly different between Zones 1 and 3 (one-way ANOVA, corrected P = 0.04), *NK4A214* abundance was significantly different between Zones 1 and 2 (bootstrapped ANOVA, P = 0.01), and *RF39* abundance was significantly different between Zones 1 and 2 (bootstrapped ANOVA, P = 0.02) and Zones 2 and 3 (bootstrapped ANOVA, P = 0.003).

*RF39* showed significantly different mean abundance across zones when analyzed across all herbivore species (bootstrap one-way ANOVA F = 3.12, corrected P = 0.05, 3000 bootstraps) and within bovid species (bootstrap one-way ANOVA F = 4.25, corrected P = 0.05, 2000 bootstraps). Mean abundance of *RF39* specifically differed between Zones 1 and 2 (P = 0.02 for all herbivore species; P = 0.02 for only bovid species) and Zones 2 and 3 (P = 0.04 for all herbivore species; P = 0.003 for only bovid species; Fig 5). Mean *Alistipes* abundance also differed significantly among zones within bovid species (bootstrap ANOVA F = 4.12, corrected P = 0.05, 2000 bootstraps), specifically between Zones 1 and 2 (P = 0.007; Fig 5). Similarly, *Christensenellaceae*_*R-7 group* showed significant differences in mean abundance across zones when analyzed within bovid species (one-way ANOVA F = 3.06, df = 2,200, corrected P = 0.05), specifically between Zones 1 and 3 (P = 0.04; Fig 5). Lastly, *NK4A214* abundance was also significantly different between zones when analyzed within bovid species (bootstrap one-way ANOVA F = 3.45, corrected P = 0.05, 2000 bootstraps), specifically between Zones 1 and 2 (P = 0.01; Fig 5).

When analyzed across waterhole sites, mean *Christensellaceae_R-7 group* abundance was significantly different when subset for only bovid species (one-way ANOVA F = 2.28, df = 12,124, corrected P = 0.04). However, Tukey’s post-hoc test with adjusted p-values revealed no significant pairwise differences between waterhole sites.

### Alpha diversity

**Fig 6:**
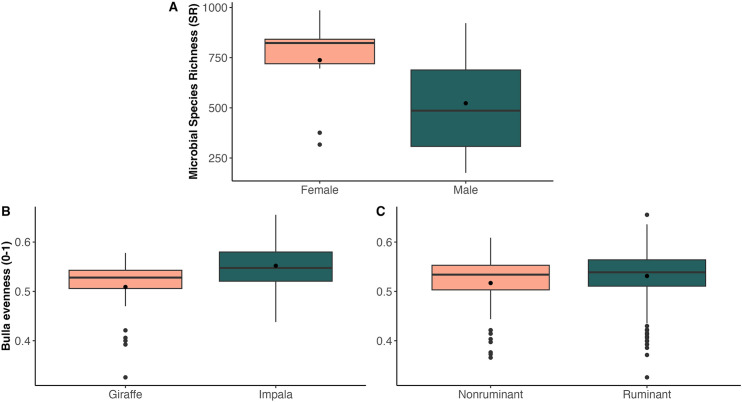
Gut microbiome alpha diversity is significantly different by sex, host species, and gut morphology. Boxplots comparing two gut microbiome alpha diversity metrics for 11 African herbivore species. Sampled species included African elephants (*Loxodonta africana*), Burchell’s/plains zebras (*Equus quagga burchellii*), Hartmann’s/mountain zebras (*Equus zebra hartmannae*), Angolan giraffes (*Giraffa camelopardalis angolensis*), common eland (*Taurotragus oryx*), kudu (*Tragelaphus strepsiceros*), black-faced impala (*Aepyceros melampus petersi*), springbok (*Antidorcas marsupialis*), gemsbok/oryx (*Oryx gazella*), blue wildebeest (*Connochaetes taurinus*), and red hartebeest (*Alcelaphus buselaphus*). a) Microbial species richness (SR) was significantly more abundant in female eland than males (permutation t-test, 2000 reps, corrected P = 0.03). b) Mean Bulla evenness was significantly greater in impala (0.552) than giraffes (0.509; one-way ANOVA, Tukey’s post-hoc corrected P = 0.02). c) Mean Bulla evenness was weakly but significantly greater in ruminants (0.531) than nonruminants (0.517; bootstrapped t-test, corrected P = 0.03).

We did not detect significant differences in mean Shannon diversity, inverse Simpson’s diversity, or Faith’s PD among herbivore species, between sexes, across zones, between ruminants and nonruminants, among feeding guilds, or among waterhole sites. However, when microbial richness was calculated alone, we found that richness was significantly greater in females than males among all herbivore species (two-tailed t-test, corrected P = 0.04) and within only bovid species (two-tailed t-test, corrected P = 0.03). To determine which herbivore species may be driving this trend, we conducted t-tests separately for each herbivore species by sex and found that eland were the only species where microbial richness was significantly greater in females than males (permutation t-test, 2000 reps, corrected P = 0.03; Fig 6a).

We did not detect significant differences in the means of four of the five evenness indices (i.e., Camargo, Pielou, Simpson, E_var_) among herbivore species, between sexes, across zones, between ruminants and nonruminants, among feeding guilds, or among waterhole sites. Mean Bulla evenness was significantly different among herbivore species (one-way ANOVA F = 2.26, df = 10,301, corrected P = 0.02); a post-hoc test revealed that this was specifically driven by the pairwise interaction of giraffes (mean Bulla evenness = 0.509) and impala (mean Bulla evenness = 0.552; Tukey’s post-hoc corrected P = 0.02; Fig 6b). Additionally, mean Bulla evenness was weakly but significantly higher in ruminants (0.531) than nonruminants (0.517; bootstrapped t-test, corrected P = 0.03; Fig 6c). Mean Bulla evenness was not significantly different between sexes, across zones, among feeding guilds, or among waterhole sites.

### Beta diversity

**Fig 7:**
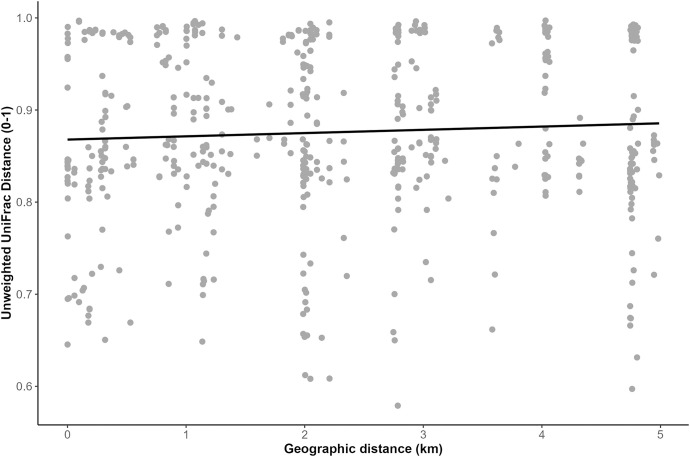
Unweighted UniFrac distance is positively correlated with geographic distance between elephant samples. Unweighted UniFrac dissimilarity correlates weakly (Mantel statistic r = 0.07) but significantly (p = 0.05) to geographic distance (in km) between pairwise elephant (*Loxodonta africana*) samples (n = 33) in Etosha National Park, Namibia. Each point represents the pairwise difference between two elephant samples, thus the total number of points is 33x33.

Beta diversity analyses did not result in any significant differences in weighted or unweighted UniFrac distances among herbivore species, between sexes, among zones, between ruminants and nonruminants, among feeding guilds, or among waterhole sites. When we compared host-specific UniFrac distances against host-specific geographic distances, elephants were the only species that showed a weak but significant relationship between unweighted UniFrac dissimilarity and geographic distance between samples (Mantel statistic r = 0.07, p = 0.05; Fig 7). There were no significant relationships between weighted UniFrac and geographic distance between samples within any herbivore species.

## Discussion

We characterized GMB diversity and community structure within and across 11 African herbivore species based on intrinsic and extrinsic variables. Three main drivers of microbial diversity and community structure—host species, environment, and diet—likely favored distinct microbial taxa that perform specialized functions in their respective hosts. Additionally, the weak but significant Mantel test results presented a unique case study in elephants highlighting the potential importance of social behavior in evaluating microbiome differences across species. Collectively, these results offer exploratory insights into the microbial framework that shapes host health, behavior, and adaptation in wildlife communities that inhabit semiarid environments.

Our average sample size of ~28 fecal samples per species (and in the case of Hartmann’s zebras, as low as 11 samples) limited our ability to make definitive conclusions about microbial diversity and structure for these herbivore species as a whole. Sample sizes were further reduced when accounting for differences by waterhole site, which limited our statistical power for these analyses. Future microbiome analyses in ENP and/or with these same host species should prioritize collecting greater sample sizes for species, age, and location to support more accurate interpretations of these variables. Additionally, previous research has cautioned using fecal samples to characterize the entire GMB of mammalian hosts due to differences in microbial diversity between different regions of the gastrointestinal tract [77,78]. However, analyzing microbial communities in the feces still offers important applications for host health. For example, changes in microbial load in the feces is associated with disease in hosts [79], and fecal microbiota retained better dietary signals than intestinal flora [80]. Importantly, the convenience and noninvasive nature of collecting fecal samples allows wildlife managers to incorporate long-term monitoring of host-associated microbial taxa, which has global implications for wildlife conservation and ecosystem health.

We analyzed core microbial taxa detected in ≥50% of individuals across and within herbivore species as opposed to the ≥ 90%, ≥ 80%, or ≥75% levels based on assumptions of drivers of microbial diversity in host species and limitations with taxon sample sizes. A key takeaway from this approach is the importance of identifying core microbial taxa both within host species as well as across a community of hosts, as these dual perspectives provide distinct and important insights for noninvasive monitoring of wildlife health. For example, changes in core microbial taxa may signal a decline in host health and/or impacts of environmental perturbations [81]. Identifying baseline profiles of herbivore species’ GMBs is crucial to enable detection and forecasting of microbial community shifts in the future, especially as ENP’s climate and plant communities continue to change [38,82].

Host species may exert distinct selective pressures on microbial diversity based on different life history traits. Microbial richness was significantly greater in female eland than males (Fig 6a), and sex-specific differences in relative abundance were observed in the core genera *Christensenellaceae*_*R-7 group* (across all herbivores; Fig 3c) and in *P-251-O5* (within gemsbok only; Fig 3d). Sex hormones such as estrogen and androgen can modulate GMB composition [83] resulting in sexual dimorphism in host GMBs, even if the host species themselves do not exhibit obvious sexually dimorphic traits. Other mammal species including mink (*Neovison vison*) [14] and northern elephant seals (*Mirounga angustirostris*) [84] have been characterized as having distinct GMB community structures between males and females.

The presence or absence of core microbial taxa in host species may indicate specialized functional niches that complement hosts’ unique diets, physiologies, and habitat requirements [19,29,85]. For example, ≥ 50% of elephants contained *Bacteroidales_RF16 group* and *WCHB1–41*, neither of which occurred in the majority of samples from other herbivores. *Bacteroidales_RF16* group is negatively associated with inflammatory biomarkers in horses [86], and other members of the phylum *Bacteroidetes* help alleviate inflammation in rodents [87,88], suggesting that elevated levels of this genus may similarly decrease inflammation in elephants as well. *WCHB1–41* thrives in anoxic environments [89], indicating that they are well adapted to elephants’ complex hindguts and long colons [1,90,91]. Additionally, *WCHB1–41* promotes biosynthesis pathways that allow hosts to efficiently extract essential nutrients from scarce, low-quality forage in harsh environments [92]. This functional niche likely bolsters elephant foraging efficiency in ENP’s semiarid landscape, as elephants are mixed foragers who subsist on a range of food resources that vary in season and quality [93,94]. Thus, decreased abundance of both *Bacteroidales_RF16* and *WCHB1–41* in elephants could signal the onset of disease or gastrointestinal disturbance.

In contrast, the core genera *Clostridia_UCG-014* and *Clostridia_VadinBB60,* which occurred in ≥50% of all herbivore samples, were not as prevalent in Burchell’s zebra, kudu, eland, and springbok (S1 Table). The class *Clostridia* performs many essential services for hosts including butyrate production [95], pathogen resistance [96,97], and anti-inflammatory functions [98]. However, *Clostridia_VadinBB60* has also been correlated with reduced cognitive function in aging mice [99], and *Clostridia_UCG-014* is associated with gut barrier dysfunction in humans [100]. This suggests that an increased abundance of specific Clostridia members may indicate illness or senescence in herbivores. Additional sampling across hosts of varying health/disease states and life stages could identify more comprehensive indicator taxa to noninvasively monitor wildlife wellbeing.

Herbivores’ GMB community structure is likely shaped more by species-specific diets than by any other intrinsic or extrinsic factor. Four core genera (*Ruminococcus*, *Gastranaerophilales*, *Treponema*, and *P-251-O5*) were found primarily in ruminant host species, collectively aiding in degrading fibrous plant material in the rumen and breaking down complex plant polysaccharides [101–106]. Henderson et al. [29] postulated that ruminants host diverse, functionally redundant microbial taxa that interact frequently to perform the same digestive functions (e.g., propionate production) depending on species-specific diet. This pattern may also explain the higher microbial evenness observed in ruminants than nonruminants (Fig 6c). While ruminants have evolved a complex stomach to facilitate maximal microbial fermentation of cellulose, nonruminants rely on different microbial taxa to degrade distinct dietary compounds via hindgut fermentation. For example, *Monoglobus,* a genus that degrades the complex plant polysaccharide pectin [107], was significantly more abundant in nonruminants than ruminants (Fig 3b).

In addition to gut morphology, dietary variability of herbivores within different feeding guilds may play a significant role in shaping GMB diversity among herbivore species. We found that giraffes (browsers) and impala (mixed foragers) drove the significant difference observed in microbial evenness among all herbivore species, with impala GMBs exhibiting higher mean evenness than giraffes. Pansu et al. [17] found that browsers and mixed foragers showed high dietary niche separation, and impala specifically had high inter-individual dietary variability. In contrast, giraffes’ more specialized diet of shrubs and trees (with preference toward *Acacia* spp.) [108,109] in ENP may drive selection for novel microbial taxa in the gut [110] and overall lower GMB diversity. Giraffes in other regions of Africa also show patterns of low microbial diversity compared to other herbivore species [12,32]. Therefore, dietary and gut morphological differences across herbivore species may significantly influence the presence and relative abundance of specialized microbial taxa. As climate change induces plant community shifts in dryland environments like ENP [38,82,111], further exploration of species-specific dietary impacts on herbivore GMBs should remain a priority in wildlife management decisions and conservation planning.

The relative abundance of bovid species’ core genera *Alistipes, NK4A214, RF39*, and *Christensenellaceae*_*R-7 group* differed significantly across ENP zones (Fig 5). Each of these genera are important to ruminant digestive efficiency and health benefits for hosts. The numerous functions of these genera include butyrate and propionate production (e.g., *Alistipes* [98], *NK4A214* [105]) and lipid metabolism (e.g., *Alistipes* [112]), and they are generally associated with healthy lifestyles in humans (e.g., *RF39* [113], *Christensenellaceae* [114]). Across all herbivore species in ENP, the core phylum *Verrucomicrobiota* also significantly differed in abundance across zones (Fig 3a); Osborne et al. [25] hypothesized that differences in *Verrucomicrobiota* abundance between two camel species may result from variations in temperature, diet, or soil microbes in each camel’s respective habitat. These five core taxa may be environmental indicators in ENP’s herbivore community that fluctuate in response to microhabitat differences and host health. For example, Zone 2 of ENP – where many bovid species exhibit a peak or trough in relative abundances of the indicator taxa mentioned above (Fig 5) – contains an area of productive soils and the most diverse vegetation communities in the park [34,38]. Varying levels of plant diversity in different ecosystems have been shown to influence GMB community composition in herbivores [22], which may explain fluctuating microbial taxa abundances in ENP’s bovid community compared to Zones 1 and 3. Additionally, Zone 1 encompasses the western, most arid region of the park (Fig 1) and receives less annual rainfall than the eastern zones [34,38]. In the dry season, herbivores’ GMB structure may shift in response to more diversified diets [12], suggesting that microbial membership varies in response to both habitat and diet, separately from host-specific selection. Overall, differences in microbiome structure in herbivores across ENP suggests that GMB plasticity could facilitate adaptations to habitat perturbations and environmental conditions [23,115].

Elephants were the only species to exhibit significant variation in microbial membership with increasing geographic distance in ENP (Fig 7). Elephants frequently engage in physical touch for social bonding [116,117], more so than other herbivores, which likely increases microbial transmission among herd members and could heighten observed GMB differences between herds that interact infrequently or not at all. Mammalian GMB diversity can be shaped by direct and indirect social transmission of both pathogenic [118–120] and potentially commensal/mutualistic microbes [121]. Indirect microbial transmission between hosts has been documented both within host species (e.g., baboons) [122] and across host species (e.g., chimpanzees and gorillas) [35]. This ‘social microbiome’ [121] is an under-studied area of microbial ecology [123] and has implications for host health not just within species, but across entire wildlife communities. Wildlife managers should consider the importance of host-associated taxa and social behaviors for conservation planning to preserve core microbes that may facilitate crucial wildlife adaptations. For instance, Huang et al. [124] found that identifying key microbial groups in giant pandas (*Ailuropoda melanoleuca*) was useful for monitoring changes to pandas’ GMB profiles over time, as a proxy for reintroduction success in the wild. Elephants in ENP represent an exciting opportunity to monitor long-term GMB variation in herds and individuals in a harsh and rapidly-changing environment.

ENP faces a variety of significant conservation challenges in the 21^st^ century [38] including increased desertification [125], water loss due to rising temperatures and evaporation rates [126], and plant community shifts [82], all leading to floral and faunal biodiversity loss. In an increasingly arid environment, large herbivores may have a more difficult time thermoregulating than other wildlife clades because of their complex digestive processes and energy requirements [127,128]. This study establishes baseline data about core microbial taxonomic abundance and occurrence in ENP’s herbivore community, laying the groundwork for a potential long-term GMB monitoring system to track microbial community shifts in host species over time. In the wake of climate change and increasing desertification of Africa’s landscapes, a GMB monitoring program can help ensure the persistence of core microbial taxa necessary for host-specific physiological functions and the long-term survival of herbivore populations.

## Conclusions

GMB diversity and community structure in ENP’s herbivore community was significantly influenced by both physiological factors (i.e., sex and gut morphology) and geography, both within and across herbivore species. Our results suggest that the relative abundance and membership of core taxa in host species fluctuate based on distinct host dietary niches, fermentation strategies, resource availability, and even social behavior (e.g., in elephants). The results of this study highlight the importance of identifying specific drivers of GMB variation within host species and across wildlife communities. Investigating differences across host GMB profiles has the potential to enhance global wildlife conservation initiatives, particularly for forecasting how these host GMB profiles may change over time in the face of rapidly changing ecosystems.

## Supporting information

S1 Table Core microbial phyla and genera (occurring at ≥1% relative abundance) found in ≥50% samples collected from 11 African herbivore species, marked with an “X” if present. Sampled herbivore species included African elephants (*Loxodonta africana*), Angolan giraffes (*Giraffa camelopardalis angolensis*), Burchell’s/plains zebra (*Equus quagga burchellii*), Hartmann’s/mountain zebra (*Equus zebra hartmannae*), kudu (*Tragelaphus strepsiceros*), common eland (*Taurotragus oryx*), springbok (*Antidorcas marsupialis*), black-faced impala (*Aepyceros melampus petersi*), gemsbok/oryx (*Oryx gazella*), red hartebeest (*Alcelaphus buselaphus*), and blue wildebeest (*Connochaetes taurinus*). Herbivore species are ordered according to evolutionary relationships, with most distantly related on the left to more closely-related on the right. Core microbial taxa marked with an asterix are species-specific taxa found in specific herbivore species but not the majority of the herbivore community.
(DOCX)
