# Supplementary material for: Interspecific variation in gut microbiome diversity across the Etosha National Park herbivore community
Source: PLoS One. 2025 Oct 9;20(10):e0333639. doi: 10.1371/journal.pone.0333639 (PMC12510545; doi:10.1371/journal.pone.0333639)
Supplement: S1 Table — Sampled herbivore species included African elephants (Loxodonta africana), Angolan giraffes (Giraffa camelopardalis angolensis), Burchell’s/plains zebra (Equus quagga burchellii), Hartmann’s/mountain zebra (Equus zebra hartmannae), kudu (Tragelaphus strepsiceros), common eland (Taurotragus oryx), springbok (Antidorcas marsupialis), black-faced impala (Aepyceros melampus petersi), gemsbok/oryx (Oryx gazella), red hartebeest (Alcelaphus buselaphus), and blue wildebeest (Connochaetes taurinus). Herbivore species are ordered according to evolutionary relationships, with most distantly related on the left to more closely-related on the right. Core microbial taxa marked with an asterix are species-specific taxa found in specific herbivore species but not the majority of the herbivore community. (DOCX) [file pone.0333639.s001.docx]

**Supplementary Table 1.**

|  |  | **Elephant** | **Giraffe** | **Burchell's zebra** | **Hartmann's zebra** | **Eland** | **Gemsbok** | **Kudu** | **Red hartebeest** | **Blue wildebeest** | **Impala** | **Springbok** |
| --- | --- | --- | --- | --- | --- | --- | --- | --- | --- | --- | --- | --- |
| Core Phyla | *Bacteroidota* | X | X | X | X | X | X | X | X | X | X | X |
|  | *Firmicutes* | X | X | X | X | X | X | X | X | X | X | X |
|  | *Verrucomicrobiota* | X | X | X | X | X | X | X | X | X | X | X |
|  | *Spirochaetota** |  |  |  |  | X |  | X |  | X |  | X |
|  | *Cyanobacteria** |  | X |  |  | X |  |  |  |  | X |  |
| Core Genera | *Alistipes* | X | X | X | X | X | X | X | X | X | X | X |
|  | *Bacteroidales_RF16** | X |  |  |  |  |  |  |  |  |  |  |
|  | *Bacteroides* | X | X | X | X | X | X | X | X | X | X | X |
|  | *Christensenellaceae_R-7 group* | X | X | X | X | X | X | X | X | X | X | X |
|  | *Clostridia_UCG-014* | X | X |  | X |  | X |  | X | X | X | X |
|  | *Clostridia_VadinBB60* | X | X |  | X | X | X |  | X | X | X |  |
|  | *Coprostanoligenes* | X | X | X | X | X | X | X | X | X | X | X |
|  | *Gastranaerophilales** |  | X |  |  | X |  |  |  |  |  |  |
|  | *Lachnospiraceae_unclassified* | X | X | X | X | X | X | X | X | X | X | X |
|  | *Monoglobus* | X | X | X | X |  | X | X | X | X | X | X |
|  | *NK4A214* | X |  | X | X | X | X | X | X | X | X |  |
|  | *P-251-O5** |  |  |  |  |  | X |  |  |  |  | X |
|  | *Prevotellaceae_UCG-004* | X | X | X | X | X | X | X | X | X | X | X |
|  | *RF39* | X | X | X | X | X | X | X | X | X | X |  |
|  | *Rikenellaceae_RC9* | X | X | X | X | X | X | X | X | X | X | X |
|  | *Roseburia* | X | X | X | X | X | X | X | X | X | X | X |
|  | *Ruminococcus** |  |  |  | X |  |  | X | X | X |  |  |
|  | *Treponema** |  |  |  |  | X |  | X |  | X |  | X |
|  | *UCG-005* | X | X | X | X | X | X | X | X | X | X | X |
|  | *UCG-010* | X | X | X | X | X | X | X | X | X | X | X |
|  | *Uncultured* | X | X | X | X | X | X | X | X | X | X | X |
|  | *WCHB1-41** | X |  |  |  |  |  |  |  |  |  |  |
